# The Genetic Wiring of Plant Trichomes: From Initiation to Fate Specification

**DOI:** 10.1002/advs.202512008

**Published:** 2025-11-29

**Authors:** Meng Li, Yuanbo Huang, Yadi Du, Shuang Wu

**Affiliations:** ^1^ College of Horticulture Anhui Agricultural University Hefei China; ^2^ College of Horticulture Fujian Agriculture and Forestry University Fuzhou China; ^3^ Haixia Institute of Science and Technology State Key Laboratory of Agriculture and Forestry Biosecurity Fujian Agriculture and Forestry University Fuzhou China

**Keywords:** glandular cells, HD Zip, morphogenesis, plant development, trichome

## Abstract

Plant trichomes serve as essential physical barriers and highly specialized metabolic powerhouses, producing a vast array of ecologically and economically significant compounds. While the MYB‐bHLH‐WD40 (MBW) complex governing unicellular trichome development in *Arabidopsis thaliana* is well‐established, accumulating evidence suggests the existence of distinct mechanisms in regulating multicellular trichomes in other plant species. This review summarizes recent breakthroughs revealing significant divergence in species forming multicellular and glandular trichomes. The molecular networks orchestrating trichome fate determination and morphogenesis are focused, emphasizing the pivotal role and dose‐dependent mechanisms of homeodomain‐leucine zipper class IV (HD‐Zip IV) transcription factors. This review highlights the complexity and species specificity of trichome developmental programming. Understanding these underlying developmental blueprints is fundamental for elucidating how plants build these metabolic powerhouses and holds promise for future applications in enhancing plant defense and optimizing natural product biosynthesis.

## Graphical abstract

Plant trichomes are not just simple hairs but metabolic powerhouses shaped by complex genetic programs. While the MYB‐bHLH‐WD40 module is well studied in *Arabidopsis*, recent findings reveal that HD‐Zip IV transcription factors drive multicellular and glandular trichome development through dose‐dependent mechanisms. This review explores how diverse regulatory networks control trichome initiation, fate, and function—offering new paths for engineering plant defense and natural product synthesis.

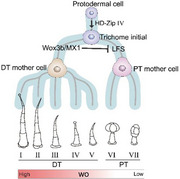

## 1 Introduction

**Figure 1:**
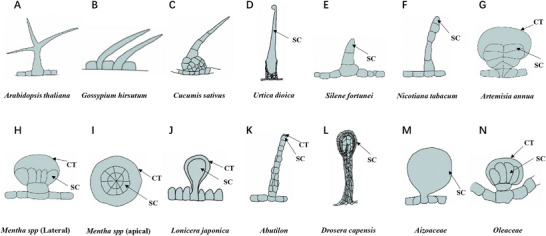
Morphological diversity of plant trichomes and the derived structures. A) Branched unicellular trichome in *Arabidopsis thaliana*. B) Unbranched unicellular cotton fiber in *Gossypium hirsutum*. C) Multicellular fruit spine in *Cucumis sativus*. D) Stinging hair in *Urtica dioica*. E) Mucilage‐secreting trichome in *Silene fortunei*. F) Glandular trichome in *Nicotiana tabacum*. G) Peltate glandular trichome in *Artemisia annua*. H,I) Lateral and apical views of peltate glandular trichomes in *Mentha spp*. J,K) Nectar‐secreting trichomes in *Lonicera japonica* and *Abutilon*, respectively. L) Digestive gland in *Drosera capensis*. M,N) Salt glands in *Aizoaceae* and *Oleaceae*, respectively. SC: secretory cell. CT: cuticle.

Most terrestrial organisms develop hair‐like structures on their epidermal surfaces.^[^ ^1^ ^]^ In animals, these structures—composed primarily of keratin‐based filaments‐serve relatively simple roles, such as thermoregulation or sensory detection.^[^ ^2^ ^]^ In contrast, plant epidermal outgrowths, termed trichomes, a word from the Greek trichoma meaning “hair,” exhibit remarkable morphological and functional diversity.^[^ ^3^ ^]^ Beyond their hair‐like appearance, plant trichomes can adopt branched, peltate, or glandular forms, often producing specialized metabolites with ecological and economic significance.^[^ ^4^, ^5^ ^]^ Trichomes vary substantially in structure and development across species. *Arabidopsis* trichomes are typically unicellular structure with three branches while cotton fibers normally develop into an extremely elongated unicellular structure (**Figure** **1**A,B). In contrast, cucumber fruit warts consist of different type of cells, carrying the silicified spine and underlying tubercules (Figure 1C).^[^ ^6^, ^7^, ^8^ ^]^

Despite their microscopic scale, trichomes as well as their animal counterparts have profoundly shaped human civilization. Animal fur and cotton fibers, specialized seed trichomes in Gossypium species, revolutionized thermal insulation, enabling human adaptation to diverse climates and fostering societal development.^[^ ^9^ ^]^ Plant trichomes have gained scientific interest due to their agricultural importance. As the primary interface between plants and their environment, trichomes play multifaceted roles in stress adaptation, including attenuating UV radiation, modulating water absorption and transpiration, mitigating heat stress, facilitating pollinator interactions, and—most critically—defending against biotic threats.^[^ ^10^ ^]^ These defensive functions rely not only on the secretion of protective compounds but also on the wide diversity of trichome morphologies that serve as physical barriers.^[^ ^11^, ^12^ ^]^

Beyond passive barriers, glandular trichomes employ sophisticated biochemical strategies, synthesizing repellent or toxic metabolites such as monoterpenes, sesquiterpenes, acylsugars, and some other compounds.^[^ ^5^ ^]^ These chemicals can not only defend against biotic threats, but help plants adapt to different environments. In some species, the glandular cells become specialized to help plants survive and reproduction. The stinging hairs of *Urtica dioica* (Figure 1D) consist of a silicified multicellular base and a single needle‐like apical cell, filled with irritant substances. Upon touched, the needle tip can pierce the skin and release toxins such as histamine and acetylcholine, providing a combined mechanical and chemical defense.^[^ ^13^ ^]^ The mucilage hairs of *Silene fortunei* (Figure 1E)^[^ ^14^ ^]^ achieve chemical defense by secreting sticky polysaccharides that deter the movement of insects. Usually the glandular cells exhibit the morphology of slightly enlarged head cells like in tobacco (Figure 1F). In some other species, the glandular head can form multicellular shield shape known as peltate trichomes such as in Artemisia and mint (Figure 1G–I). These peltate trichomes primarily synthesize volatile terpenoids, which repel animals through odor or direct toxicity.^[^ ^15^, ^16^, ^17^ ^]^ In addition to biotic stress, halophytes have evolved specialized salt glands to cope with abiotic stress. These glands actively transport salts to the leaf surface, forming a white crystalline salt layer to maintain ionic homeostasis. Typical salt gland structures include single‐celled salt bladders with an enlarged cuticular chamber (Figure 1M) and multicellular salt glands morphologically similar to peltate trichomes (Figure 1N).^[^ ^18^ ^]^

Beyond defense, some glandular trichomes have evolved other unique functions. The nectaries (Figure 1J,K) form specialized papillae, a type of multicellular glandular trichome, to secrete sugar‐rich nectar during flowering to attract pollinators.^[^ ^19^, ^20^ ^]^ Carnivorous plants go further to transform the defensive structure into “predatory organs”. The tentacle‐like glandular trichomes of sundews (Figure 1L) have a head composed of inner and outer layers of secretory cells. They actively capture prey and absorb nutrients by secreting mucilage and digestive enzymes.^[^ ^21^ ^]^

Efforts to exploit trichomes for crop improvement have persisted for decades. A classic review in the 1970s summarized investigations across plant species that highlighted strong correlations between trichome density and herbivore resistance.^[^ ^8^ ^]^ This has sparked breeding programs to introgress trichome‐related loci from wild relatives into cultivated varieties.^[^ ^22^, ^23^ ^]^ However, progress has been hindered by gaps in understanding the genetic and molecular networks governing trichome development and metabolic regulation. Recent advances in glandular trichome biology have further underscored their potential as biofactories for high‐value compounds, including pharmaceuticals and essential oils.^[^ ^5^, ^24^ ^]^ These specialized structures offer unique advantages for metabolic engineering, as their compartmentalized cells minimize fitness costs associated with toxic metabolite production.^[^ ^25^, ^26^, ^27^ ^]^ While many reviews comprehensively address trichome development and metabolism, this article focuses on recent breakthroughs and emerging insights into regulatory networks, particularly in glandular trichomes.

## 2 Trichome Initiation of Multicellular Trichomes

**Figure 2:**
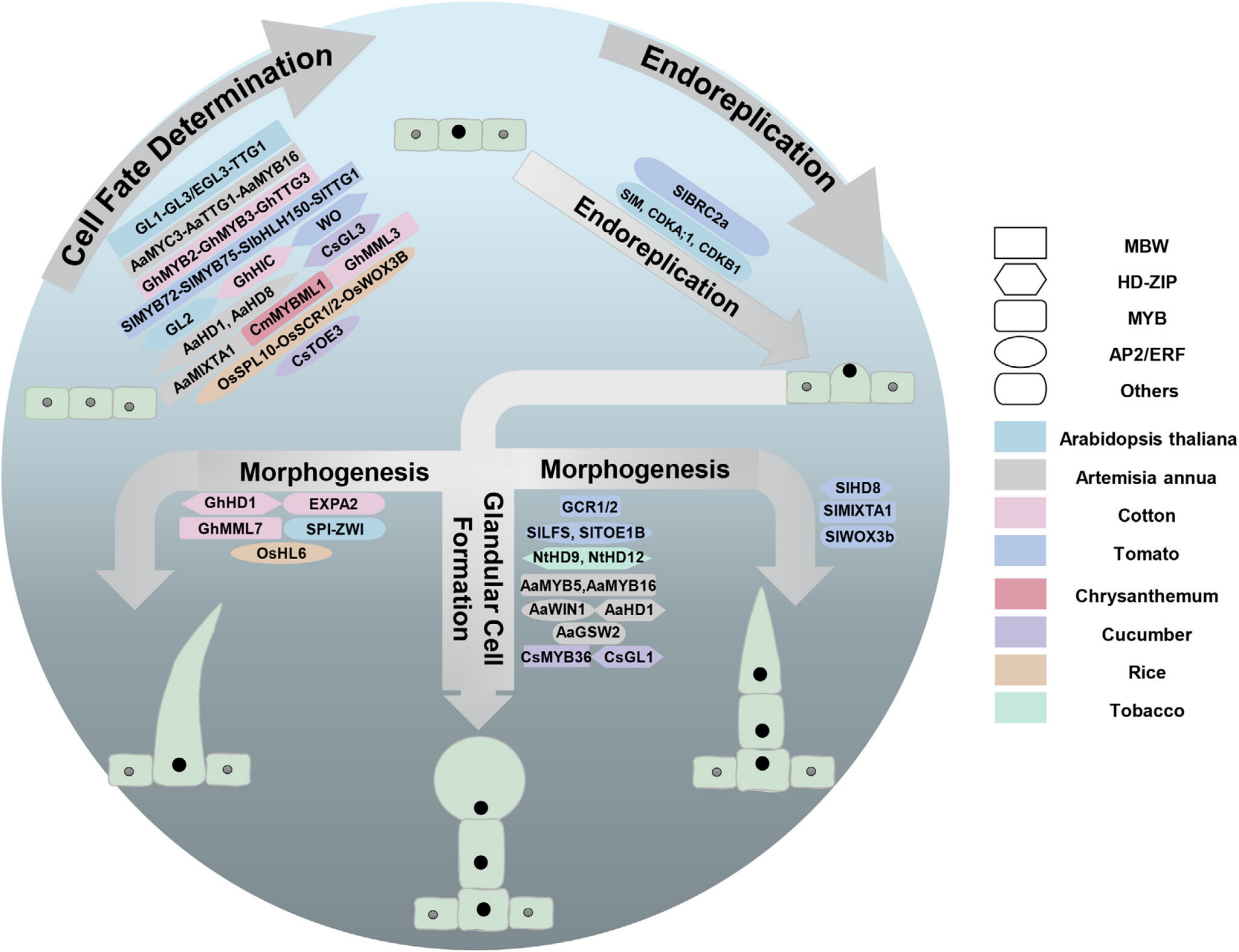
Model of trichome development. The mechanistic diagram illustrates the core molecular regulatory networks governing trichome or glandular trichome development in *Arabidopsis thaliana*, *Artemisia annua*, *Gossypium hirsutum* (cotton), *Solanum lycopersicum* (tomato), *Chrysanthemum morifolium* (chrysanthemum), *Cucumis sativus* (cucumber), *Oryza sativa* (rice), and *Nicotiana tabacum* (tobacco). Proteins belonging to the same family are represented by identical shapes, while colors indicate the corresponding plant species.

The molecular mechanisms governing trichome initiation have been extensively studied across plant species (**Figure** **2**), yet a universal model explaining how protodermal cells commit to trichome differentiation remains elusive. Emerging evidence suggests regulatory pathways diverge significantly between species, particularly between those forming unicellular trichomes such as *Arabidopsis thaliana*, and those producing multicellular structures including *Solanum lycopersicum*.^[^ ^28^, ^29^, ^30^, ^31^ ^]^ This disparity suggests the complexity of trichome developmental programming and highlights the need for species‐specific investigations.

**Figure 3:**
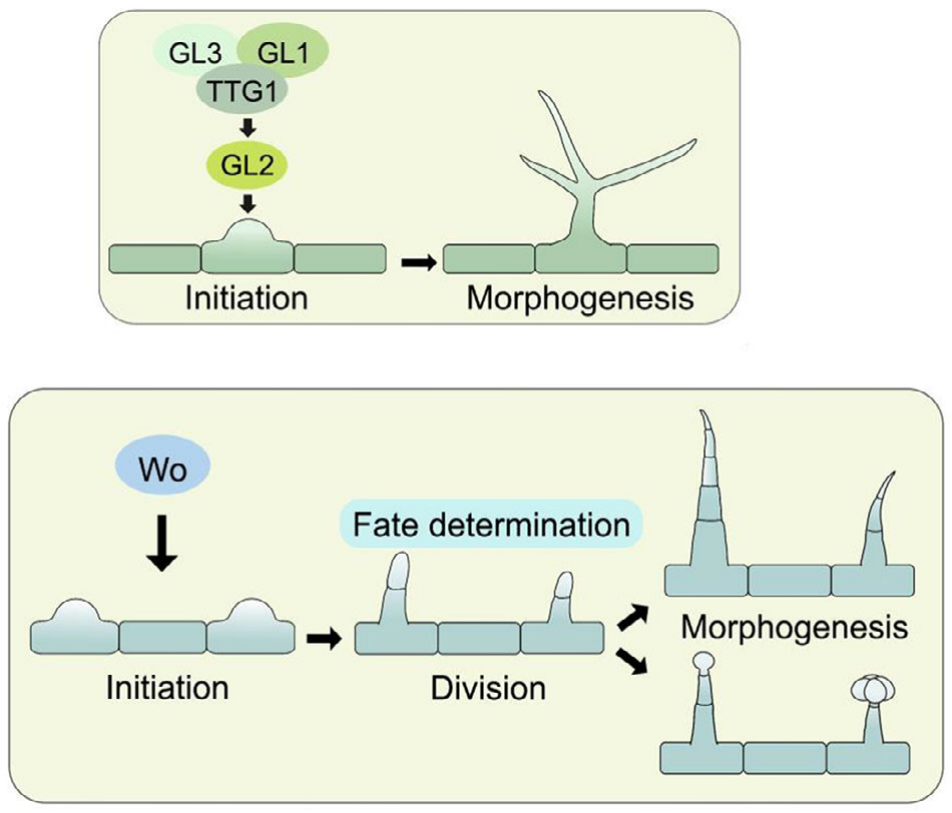
Illustration of distinct developmental processes and regulatory mechanisms between unicellular and multicellular trichomes. The upper panel depicts the well‐established regulatory model mediated by the MYB‐bHLH‐WD40 (MBW) complex in *Arabidopsis*. The lower panel illustrates the more complex processes forming different types of multicellular trichomes in tomato. Wo, instead of homologs of the MBW complex regulators, has been shown to regulate the development of multicellular trichomes in tomato.

Trichome density exhibits striking variability across plant taxa and fluctuates dynamically within species in response to environmental cues like light, herbivory, or nutrient availability.^[^ ^7^, ^32^, ^33^, ^34^, ^35^, ^36^, ^37^, ^38^ ^]^ This plasticity implies polygenic regulation, with genetic networks integrating developmental and stress signaling inputs. Early mechanistic insights derive largely from *Arabidopsis*, which develops unicellular, branched trichomes.^[^ ^18^, ^19^ ^]^ Forward genetic screens identified a core regulatory module—the MYB‐bHLH‐WD40 (MBW) complex—comprising transcription factors GLABRA1 (GL1), GLABRA3/ENHANCER OF GLABRA3 (GL3/EGL3), and TRANSPARENT TESTA GLABRA1 (TTG1) (**Figure** **3**).^[^ ^39^, ^40^, ^41^, ^42^ ^]^ This complex activates trichome initiation through HD‐Zip IV gene GLABRA2 (GL2), while suppressing neighboring epidermal cells through lateral inhibition mediated by competitive inhibitors like CAPRICE (CPC) and TRIPTYCHON (TRY), two small MYB proteins.^[^ ^43^, ^44^, ^45^ ^]^ Spatial patterning is thus established via cell‐to‐cell communication, ensuring regularly spaced trichomes to avoid clustering. This well‐established model has been well described in previous reviews.^[^ ^42^, ^46^, ^47^, ^48^ ^]^

Further studies have verified the involvement of MBW homologs in trichome development in many species, particularly within the Brassicaceae family, to which *Arabidopsis* also belongs.^[^ ^49^, ^50^ ^]^ In *Brassica villosa*, a species forming dense, unbranched unicellular trichomes on stems and leaves, a quantitative trait locus (QTL) analysis linked the expression of BoTRY with hairy or glabrous phenotypes.^[^ ^51^ ^]^ More studies have indicated that such conservation extends beyond Brassicaceae. Studies in cotton found that bHLH family transcription factor GhDEL65 physically interacts with R2R3 MYB transcription factor GhMYB2 and GhMYB3, and form a MBW complex with WD40 protein GhTTG3, which is proposed to play roles in regulating the formation of cotton fiber.^[^ ^52^ ^]^ A recent study has shown that the formation of short trichomes on Nigella petals is controlled by an MBW complex of NidaMYB5, NidaGL3, and NidaTT8. Intriguingly, the development of long trichomes on the same petals requires a simpler regulatory module, involving only NidaMYB5 and NidaGL3.^[^ ^53^ ^]^

Beyond unicellular trichomes, MBW‐mediated regulation may also operate in multicellular trichomes. In tomato, R2R3‐MYB transcription factors SlMYB72 and SlMYB75 antagonistically regulate trichome formation by competitively binding to MBW complexes with SlbHLH150 and SlTTG1, thereby modulating expression of SlCycB2, a repressor of trichome initiation (its annotation is discussed later).^[^ ^54^ ^]^

In *Artemisia annua*, the development of glandular trichomes responsible for artemisinin production is also regulated by genes associated with the MBW complex. A jasmonate‐inducible bHLH transcription factor, AaMYC3, together with the WD40 protein AaTTG1, coordinately regulates both trichome density and artemisinin biosynthesis.^[^ ^55^, ^56^ ^]^ Overexpression of AaMYC3 significantly increases trichome density and artemisinin content, whereas RNAi‐mediated silencing results in a marked reduction in both. Notably, AaMYC3 promotes glandular trichome development by directly activating AaHD1 whose homolog GhHD‐1 also regulates trichome development in cotton.^[^ ^55^ ^]^ In addition, AaMYB16 was found to interact with AaHD1 to regulate the initiation of both glandular and non‐glandular trichomes.^[^ ^57^ ^]^

These findings appear to suggest the conserved MBW mediated mechanism across species with unicellular or multicellular trichomes. However, many other findings suggest functional diversification. In tomato and other species, the MBW complex controls anthocyanin/flavonoid accumulation.^[^ ^58^ ^]^ Overexpression or enhanced expression of CPC and GL3 in tomato affected only anthocyanin, with minimal effect on trichomes.^[^ ^59^ ^]^ In addition, over‐expression of *GL1* in tobacco does not result in increased trichome formation.^[^ ^60^ ^]^ These observations imply evolutionary divergence in regulatory wiring of trichomes.

The idea of distinct mechanisms is further supported by studies of HD‐Zip IV factors, which govern multicellular trichome initiation in many non‐Arabidopsis systems. Woolly (Wo), an HD‐Zip IV gene, acts as the master regulator of trichome formation in tomato (Figure 3).^[^ ^35^, ^61^, ^62^ ^]^ Loss‐of‐function of Wo dramatically reduces trichomes, while gain‐of‐function causes a “woolly” super‐hairy phenotype.^[^ ^35^, ^61^, ^62^ ^]^ Lanata (Ln), a close Wo relative, has also been shown to regulate tomato trichome density, and gain‐of‐function mutations in Ln induce similar hairy phenotypes.^[^ ^63^ ^]^ Genetic evidence thus suggests HD‐Zip IV regulators primarily drive trichome formation, whereas the MBW mechanism is more involved in anthocyanin accumulation in tomato. Two Wo orthologs in *Arabidopsis*, ML1 and PDF2, regulate epidermal development but show no involvement in MBW‐mediated trichome initiation, which challenges the universal regulatory models.^[^ ^64^, ^65^ ^]^ Despite this, evidence obtained in most plant species points that trichome initiation associated tightly with the activity of HD‐Zip IV transcription factors. Map‐based cloning in cotton identified HD‐Zip IV transcription factor HIC as the key regulator of trichome initiation in leaves and stems.^[^ ^66^ ^]^ In cucumber, CsGL3, another HD‐Zip IV gene, has been shown to control the trichome initiation and the loss‐of‐function of this gene leads to the complete loss of trichomes.^[^ ^67^ ^]^ In Nigella petals where both short trichomes and long hairs are formed, the HD‐Zip IV gene NidaGL2 is found to be important for the formation of both trichome types.^[^ ^53^ ^]^ Notably, the HD‐Zip I gene NidaLMI1 appears to be specifically involved in the formation of short trichomes, and  suggests that NidaLMI1 acts upstream of MBW complex.^[^ ^53^ ^]^

A recent study adds another layer of complexity by uncovering a previously uncharacterized trichome structure in tomato flowers. These interlocking trichomes play a key role in fusing neighboring anthers to form a unique floral structure, the anther cone, which is essential for the formation of cleistogamous flowers.^[^ ^68^ ^]^ Interestingly, these interlocking trichomes are unicellular, unlike the multicellular trichome structures formed in other tomato tissues.^[^ ^68^ ^]^ Nevertheless, the same set of HD‐Zip regulators appear responsible for initiating both types of trichomes, with Wo primarily involved in conventional multicellular trichomes formation, and HD7 and HD7‐like, two close homologs of Wo, primarily involved in forming interlocking trichomes.^[^ ^68^ ^]^

Beyond MBW and HD‐Zip genes, other gene families also influence trichome development. CsTOE3, a APETALA2/ethylene‐responsive factor (AP2/ERF) transcription factor, was found to regulate the initiation of cucumber fruit warts and disruption of this gene function causes wart‐less fruits, while the trichomes in other tissues are not affected.^[^ ^69^ ^]^ In addition to fruit warts, another specialized trichome, cotton fiber, is controlled by a MIXTA transcription factor, MYB25‐like (GHMML3) and loss‐of‐function of this gene generate fiber‐less seeds.^[^ ^70^ ^]^ Further evidence indicates that activation of GHHD‐1 and GHMYB25 can promote cell fate to fibers in cotton seeds.^[^ ^71^ ^]^ In monocot rice, trichome formation appears to be controlled by a quite different mechanism. Knockout of OsSPL10 leads to the disappearance of trichomes in rice, and the regulatory model comprising OsSPL10‐OsSCR1/2‐OsWOX3B is proposed to play key role in trichome initiation in rice.^[^ ^72^ ^]^ In maize, another monocot species, trichomes are also controlled by SPL family members.^[^ ^73^ ^]^ Three maize SPL transcription factors (ZmSPL10/14/26) target and activate ZmWOX3A, which is speculated to control the trichome initiation via auxin pathway.^[^ ^73^ ^]^ The similar phenotype between *zmspl10/14* double mutant and *osspl10* suggests a highly conserved regulation in these two monocot species.

In *A. annua*, the MADS‐box factor AaSEP1 enhances glandular trichome density by promoting the AaHD1‐AaMYB16 network which regulates initiation via jasmonic acid (JA) signaling.^[^ ^74^ ^]^ This MYB‐HD Zip regulatory model is supported by further evidence showing that AaMIXTA1and AaHD8 form protein complex to promote trichome initiation in *A. annua*.^[^ ^75^ ^]^ Interestingly, CmMYBML1, the homolog of AaMIXTA1 in chrysanthemum, has also been found to positively regulate trichome initiation.^[^ ^76^ ^]^ In tomato, auxin‐induced SlARF4 increases trichome density by directly inhibiting R2R3‐MYB genes SlTHM1 and SlMYB52.^[^ ^77^ ^]^ In addition to transcription factors, an epidermis‐specific glutaredoxin named Hairy (H) has been cloned and the location of H expression decides the trichome distribution pattern in Antirrhinum. Interestingly, mutations of this gene appear to be involved in the evolutionary divergence of alpine and lowland *Antirrhinum* lineages.^[^ ^78^ ^]^

While these insights rely heavily on gain‐of‐function and in vitro assays, systematic genetic and loss‐of‐function analyses are needed to clarify functional conservation across species.

## 3 Fate Determination of Trichome Subtypes

Trichome morphology and function exhibit striking diversity across plant species, with distinct types often coexisting on a single plant or even within the same tissues.^[^ ^79^ ^]^ These trichome types display highly varied organization, morphology, and functions. In tomato (*Solanum lycopersicum*), seven trichome types (I–VII) are classified by structure and secretory activity: Types I and IV are glandular and produce defensive metabolites including acyl sugars, while Types VI and VII are short glandular trichomes specialized for exudate storage such as terpenoids.^[^ ^62^, ^80^ ^]^ A critical unresolved question is when and how trichome precursor cells commit to specific developmental trajectories. Lineage‐tracing studies using a specific type I/IV gland marker SlAT2 (Solyc01g105580) which encodes an acyltransferase enzyme reveal fate determination as early as the two‐cell stage, indicating that transcriptional programs specifying trichome identity activate shortly after initiation.^[^ ^81^ ^]^

**Figure 4:**
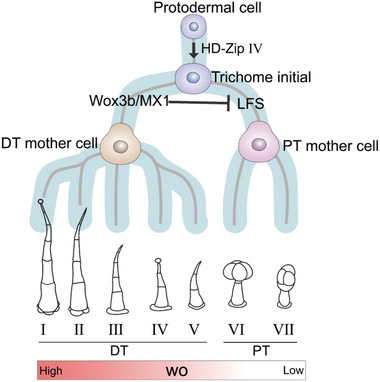
Schematic diagram showing the progressive fate determination of multicellular trichomes in tomato. When the Wo protein level is high, trichome initial cells preferentially activate a regulatory module comprising SlWox3b and MX1, promoting the fate of long digital trichomes and repressing LFS expression to block the development of short glandular trichomes. When Wo levels drop, LFS expression increases, promoting trichome initial cells to develop into short glandular trichomes.

**Figure 5:**
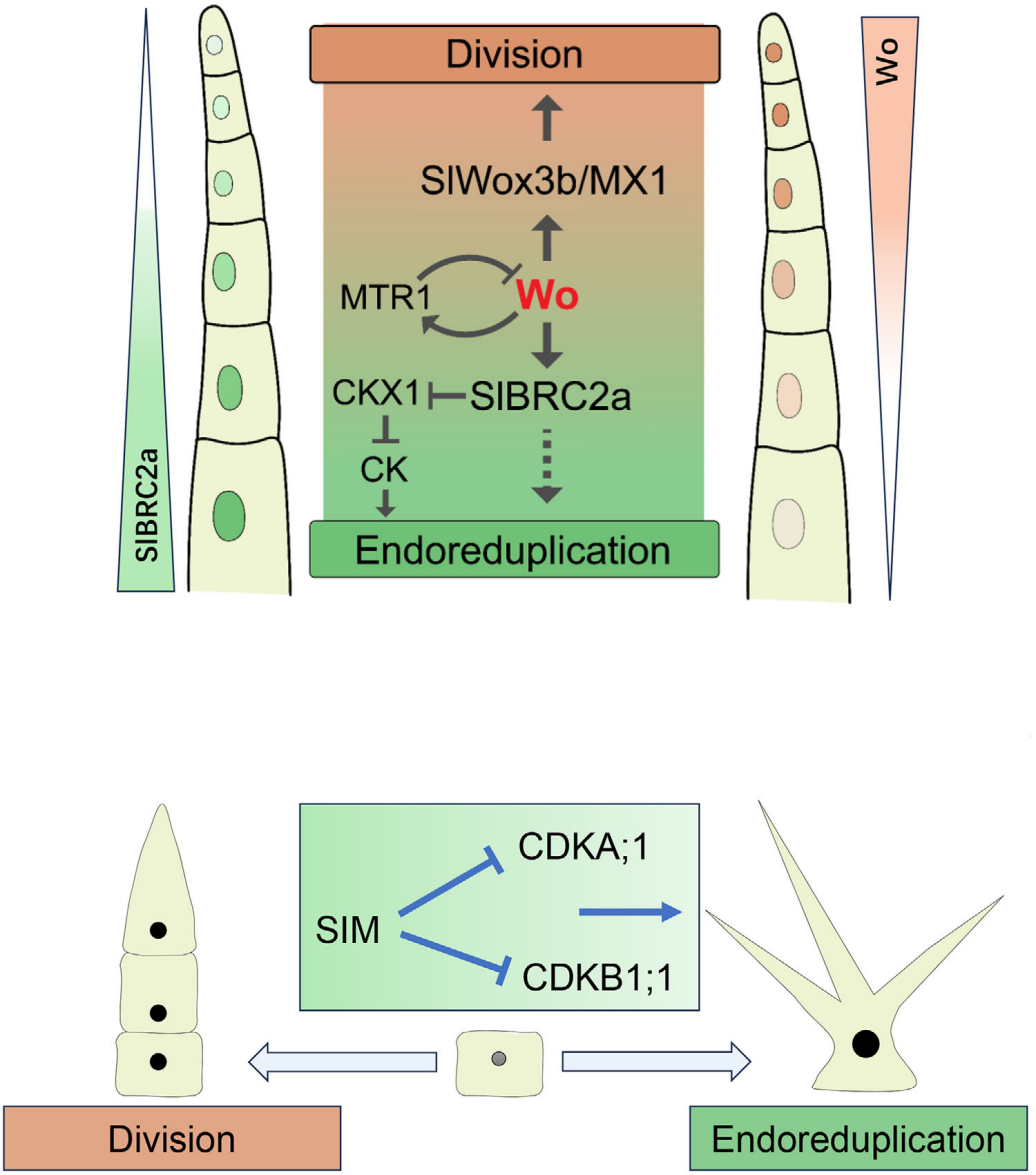
Working model depicting the establishment of morphogenesis in multicellular trichomes of tomato and *Arabidopsis* single cell trichomes. Cell division and endoreduplication occur polarly, localized to apical and basal cells, respectively, during development. This polarization is established through opposing protein gradients along the trichome axis, with the Wo protein highest in apical cells and the SlBRC2a protein highest in basal cells. High Wo protein levels promote cell division in apical cells by activating the expression of SlWox3b and MX1. High levels of SlBRC2a protein in basal cells promote endoreduplication and cell expansion, partly by enhancing cytokinin accumulation. The level of Wo is maintained by a feedback loop, where Wo activates downstream MTR expression, which in turn inhibits Wo protein accumulation, preventing excessive apical cell division. In *Arabidopsis*, SIM promotes endoreplication by binding to and inhibiting CDKA;1 and CDKB1;1.

A recent study uncovered a dose‐dependent mechanism orchestrated by Woolly (Wo) transcription factors, which biases trichome progenitor cells toward distinct developmental paths.^[^ ^62^ ^]^ In this model, higher Wo levels promote differentiation into long digitate trichomes, while low Wo levels favor short glandular trichome formation (**Figure** 4).^[^ ^62^ ^]^ This binary switch relies on an antagonistic mechanism comprising Wo self‐activation and a negative feedback loop mediated by its downstream factor, multicellular trichome repressor (MTR) (**Figure** **5**).^[^ ^62^ ^]^

This circuit generates high or low Wo levels, which bias transcriptional activation toward two antagonistic cascades leading to different trichome types.^[^ ^62^ ^]^ High Wo levels directly activate a regulatory module involving WOX3B (a WUSCHEL‐related homeobox gene) and MIXTA (a MYB‐family transcription factor), which jointly suppress short glandular trichome formation by inhibiting the AP2‐family regulator LEAFLESS (LFS) (Figure 4).^[^ ^62^ ^]^ Conversely, reduced Wo derepresses LFS, triggering glandular differentiation (Figure 4). This dosage‐sensitive mechanism creates a bistable switch ensuring mutually exclusive trichome subtypes.^[^ ^62^ ^]^ Supporting this model, wox3b/mixta double mutants convert all long digital trichomes to glandular types, while LFS overexpression hyper‐accumulates glandular trichomes.^[^ ^62^ ^]^

MTR is a downstream target of Wo and was initially annotated as SlCycB2, a designation based on early tomato genomes prone to gaps and mis‐annotation.^[^ ^61^ ^]^ This gene has been widely regarded and cited as a cell cycle regulator due to its nomenclature. However, updated sequence analysis reveals no homology to canonical cyclin B genes; instead, MTR encodes an EAR (EM) domain and a RING‐like (RM) domain, motifs typically associated with E3 ubiquitin ligases.^[^ ^62^ ^]^ Point mutagenesis demonstrated that the RM domain is essential for MTR activity.^[^ ^62^, ^82^ ^]^ Given that MTR physically interacts with Wo and elevated MTR levels dramatically degrade Wo protein while MTR mutants accumulate Wo, MTR is proposed to act as a non‐canonical E3 ligase.^[^ ^62^, ^82^ ^]^ Notably, MTR homologs regulate trichome formation beyond tomato, including in tobacco, soybean, cotton, and *Artemisia annua*.^[^ ^82^, ^83^, ^84^, ^85^ ^]^

This dose‐dependent regulation contrasts with the canonical trichome initiation pathway in *Arabidopsis thaliana*. In *Arabidopsis*, the MBW (MYB‐bHLH‐WD40) complex cell‐autonomously activates GLABRA2 (GL2), an HD‐ZIP IV gene, exclusively in trichome precursor cells.^[^ ^43^ ^]^ Spatial patterning depends on lateral inhibition rather than protein concentration gradients, precluding significant dosage effects.^[^ ^44^, ^45^ ^]^ Nevertheless, emerging evidence hints at broader functional versatility within the HD‐ZIP IV family. For example, AtML1 (*Arabidopsis thaliana* MERISTEM LAYER 1), a GL2 paralog, regulates epidermal patterning in sepals via a fluctuation‐driven, dose‐dependent mechanism. During sepal development, stochastic variations in AtML1 protein levels across epidermal cells determine cell fate, with cells exceeding a critical threshold undergoing endoreduplication to form giant cells.^[^ ^86^ ^]^ This demonstrates that HD‐ZIP IV proteins can employ dosage sensitivity to pattern tissues in *Arabidopsis*, although in a developmental context distinct from trichomes.

The dichotomy between Wo‐mediated dosage effects in tomato trichomes and GL2‐dependent cell specificity in *Arabidopsis* highlights functional divergence within the HD‐ZIP IV family. While Wo operates as a quantitative regulator, GL2 functions more as a binary switch, reflecting evolutionary adaptations to distinct developmental demands. Whether dose‐dependent mechanisms influence trichome patterning in other species, particularly those with multicellular or glandular trichomes, remains an open question.

## 4 The Morphogenesis of Multicellular Trichomes

Following fate commitment, trichome subtypes diverge through distinct cell division and cell expansion patterns to generate different morphologies.^[^ ^87^ ^]^ The branched trichomes of *Arabidopsis thaliana* have long served as a paradigm for studying trichome morphogenesis.^[^ ^29^, ^88^ ^]^ As in many other plant cells, the cytoskeleton, comprising actin filaments and microtubules, plays a central role in directing trichome cell shape in *Arabidopsis*, and this cytoskeleton‐based regulation also extends to multicellular trichomes like those in tomato.^[^ ^89^, ^90^ ^]^ Recent advances in live‐cell imaging and genetic analyses have revealed intricate spatiotemporal dynamics of cytoskeletons in tomato trichomes. Throughout development, actin and microtubules exhibit stage‐specific patterning, transitioning from transverse to spiral or longitudinal orientations to guide trichome growth dynamics.^[^ ^90^ ^]^ Genetic analyses further underscore the essential role of the SCAR/WAVE‐ARP2/3 pathway in directing trichome shapes in tomato.^[^ ^90^ ^]^

Beyond cytoskeletal regulation, multicellular trichome morphogenesis integrates coordinated cell division and expansion. In tomato, apical trichome cells undergo repeated divisions, while basal cells exit the cell cycle to undergo endoreduplication and expansion, establishing developmental polarity.^[^ ^87^ ^]^ A recent work found that Wo protein forms a gradient along the trichome axis, with highest levels in apical cells promoting division and lowest levels in basal cells triggering endoreduplication (Figure 5).^[^ ^87^ ^]^ Interestingly, SlBRC2a, a Teosinte branched1/Cincinnata/proliferating cell factor (TCP) transcription factor, establishes an opposite gradient to Wo, exhibiting higher levels in basal cells where it promotes endoreduplication via the cytokinin pathway (Figure 5).^[^ ^87^ ^]^ Such spatially restricted transcriptional programs orchestrate the transition from proliferative to expansive growth phases in tomato trichomes. In *Arabidopsis*, forcing cell division into endoreduplication is controlled by cell cycle regulators SMR and SIM, which inhibit the activity of CDKA;1and CDKB1;1 (Figure 5).^[^ ^91^ ^]^ Double mutants of these two genes exhibits a multicellular trichome phenotype in *Arabidopsis*.^[^ ^92^ ^]^ Interestingly, transformation of SMR gene from moss into *Arabidopsis* can re scue the multicellular trichome phenotype, suggesting the high conservation of this mechanism.^[^ ^93^ ^]^ However, not all trichome type structures require endoreduplication. Cells in cucumber fruit wart do not enter endoreduplication, and instead, the wart‐like growth relies on continuous cell division regulated by protein complex composed of CsGL1–CsSBS1–CsTTG1.^[^ ^94^ ^]^

In apical cells of tomato trichomes, high Wo levels are thought to activate the WOX3B/MIXTA‐mediated pathway to promote division.^[^ ^62^, ^87^ ^]^ Consistent with this, enhanced Wo levels in basal cells also cause supernumerary divisions, suggesting a strong correlation between high Wo concentration and cell division.^[^ ^87^ ^]^ Since high Wo levels preferentially activate WOX3B/MIXTA during fate determination, these same regulatory cascades may promote both trichome initiation and subsequent cell division.^[^ ^62^ ^]^ However, the function of WOX3B in rice appears to be more diversified. OsWOX3B is activated by the OsSPL10–OsSCR1/2 regulatory module and functions in trichome initiation. It was also found that OsWOX3B promotes trichome elongation by activating the AP2/ERF transcription factor OsHL6 to promote auxin biosynthesis.^[^ ^95^ ^]^

Beyond conventional trichomes, MIXTA factors are also involved in the morphogenesis of specialized trichomes. In cotton, reduced expression of GHMML7 via RNAi leads to dramatically shorten fibers.^[^ ^96^ ^]^ In addition, nectar guide trichomes represent another specialized type with length variation between species. In *Mimulus*, the MIXTA‐like R2R3‐MYB gene GUIDELESS controls nectar guide development.^[^ ^97^ ^]^ Nonsynonymous substitutions in a conserved MIXTA‐like motif generate a recessive allele in *M. parishii* that represses trichome elongation, facilitating the transition from bumblebee pollination to selfing.^[^ ^97^, ^98^ ^]^

Accumulating evidence suggests that the MIXTA family of MYB transcription factors is widely implicated in epidermal modification across land plants. PaleoMIXTA (termed PMX in *Thalictrum*, Ranunculaceae) represents pre‐core eudicot duplications, and its ortholog TthPMX from *T. thalictroides* is found to regulate leaf trichome development in tobacco.^[^ ^99^ ^]^ Further RNA‐Seq on virus‐induced gene silencing (VIGS)‐based gene‐silenced plants suggests TthPMX modulates trichome development via microtubule‐associated pathways, indicating a potentially conserved eudicot mechanism.^[^ ^99^ ^]^

Beyond cytokinin, jasmonic acid (JA), a key hormone responsive to insect attack and wounding, plays a key role in multicellular trichome development.^[^ ^34^, ^35^, ^36^, ^100^, ^101^ ^]^ JA promotes trichome elongation in tomato by antagonizing transcriptional repression mediated by a trichome‐preferentially expressed SlJAZ4, which targets a homeodomain‐leucine zipper gene SlHD8.^[^ ^34^ ^]^ SlHD8 loss‐of‐function causes shorter trichomes and this gene directly activates cell‐wall‐loosening genes to promote trichome elongation.^[^ ^34^ ^]^ JA‐induced development also relies on two C2H2 zinc finger proteins, Hair (H) and Hair‐like (HL), that function synergistically.^[^ ^102^ ^]^ In h/hl double mutants, long trichomes nearly disappear across all tissues.^[^ ^102^ ^]^ However, it remains unclear whether this phenotype relates to fate determination or division/expansion regulation.

## 5 Molecular Mechanisms of Glandular Cell Formation

Approximately 30% of vascular plants form glandular trichomes (GTs) with specialized secretory cells.^[^ ^103^ ^]^ These apically located glandular cells function as biochemical factories, producing abundant bioactive metabolites including defensive compounds such as acyl sugars, terpenoids and nicotine; pharmaceuticals including artemisinin and cannabinoids, as well as industrial feedstocks like essential oils.^[^ ^5^, ^24^, ^26^, ^27^ ^]^ Understanding GT development is thus critical for engineering stress‐resilient crops and optimizing in planta metabolite production.

**Figure 6:**
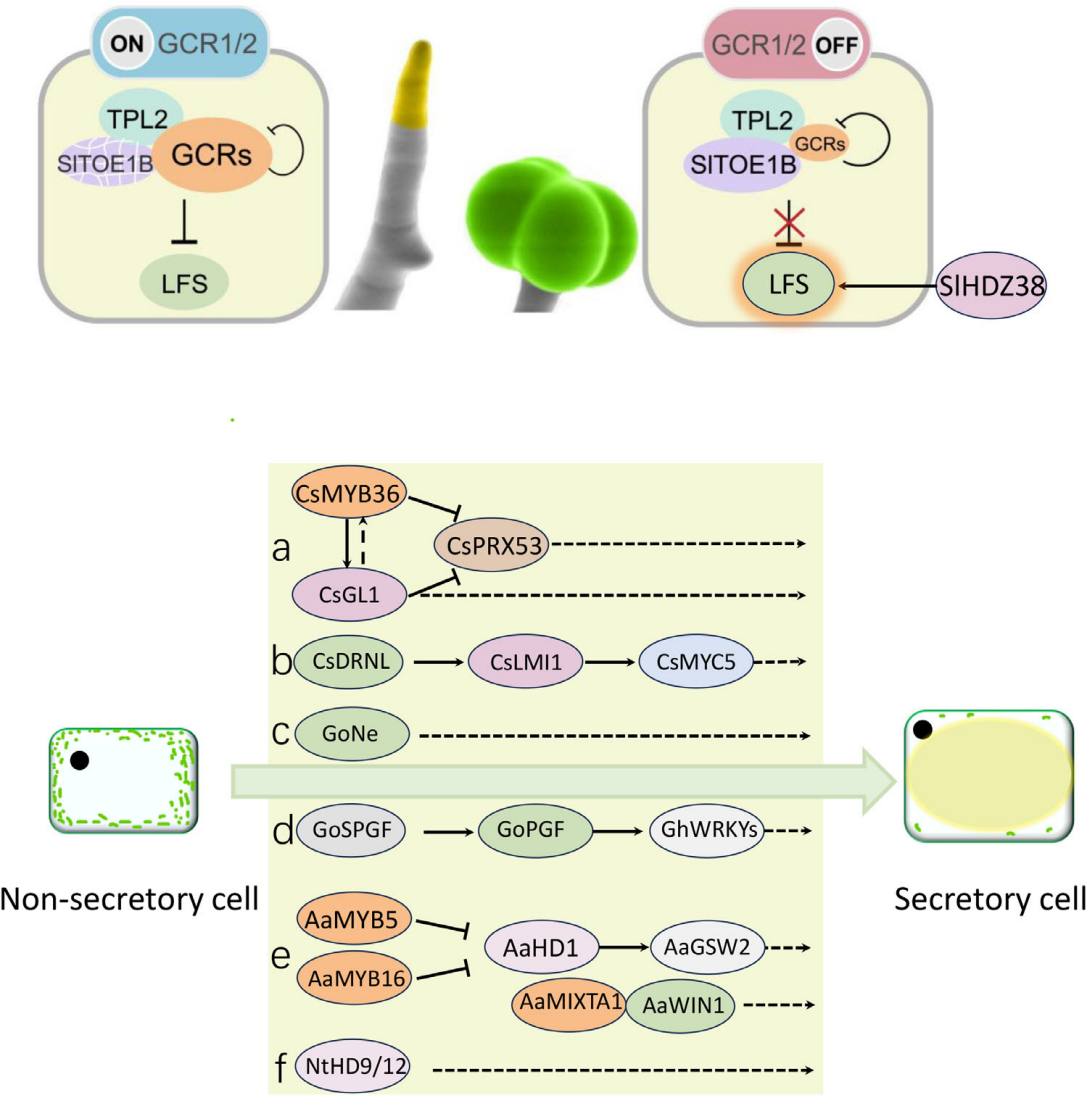
The upper panel illustrates the molecular switch controlling glandular trichome formation in tomato. Central to this switch are two MYB‐like transcription factors, GCR1 and GCR2, which are highly expressed in non‐glandular trichome tip cells but show low expression levels in glandular trichome tip cells. This spatiotemporal expression pattern of GCR1/2 is established through self‐repression and SlTOE1B‐mediated repression, wherein SlTOE1B recruits the epigenetic corepressor TPL. High expression of GCR1/2 blocks the activation of the LFS‐mediated pathway, thereby suppressing gland cell fate. The lower panel summarizes the key molecular pathways underlying gland formation in different species. Panels a to f represent cucumber glandular trichomes, citrus secretory cavities, cotton pigment glands, cotton nectar glands, Artemisia annua glandular trichomes, and tobacco glandular trichomes, respectively.

GT formation initiates through spatiotemporal fate specification in trichome apical cells.^[^ ^104^, ^105^ ^]^ A recent work reveals complex regulatory landscapes involving repressive networks controlling glandular cell formation in tomato.^[^ ^104^ ^]^ Two closely related MYB‐like transcription factors, both containing conserved SHLQMY and EAR motifs, were identified as negative regulators of glandular cell formation and thus named Glandular Cell Repressor 1/2 (GCR1/2) (**Figure** 6).^[^ ^104^ ^]^ These genes are highly expressed in apical cells of non‐glandular trichomes. Double mutants develop glandular cells on all trichomes, while GCR overexpression eliminates glandular cells.^[^ ^104^ ^]^ GCR1/2 physically interact with the AP2/ERF factor SlTOE1B, which facilitates GCR self‐inhibition by recruiting Topless (TPL)‐HDAC complexes (Figure 6).^[^ ^104^ ^]^ Notably, SlTOE1B is preferentially expressed in apical cells developing into glandular cells.^[^ ^104^ ^]^ This opposing expression pattern, combined with the inhibitory loop, creates a bistable switch determining glandular cell formation spatiotemporally. A recent study identified a positive regulator of glandular cell formation in tomato. A forward genetic screening identified a mutant named glandless, which exhibits a dramatic reduction in type VI and VII glandular trichomes became. The causative gene was cloned and encodes an homeodomain‐leucine zipper class I (HD‐ZIP I) transcription factor, SlHDZ38, suggesting it may represent an alternative pathway to the WO–WOX3b–MX1 module.^[^ ^106^ ^]^ Further supporting the diverse roles of this gene family, CsGL1—another HD‐ZIP I member—is involved in both glandular cell and non‐glandular trichome development. In citrus, knockout of *CsGL1* results in fruit surfaces retaining only papillae‐like structures. These observations indicate that while the regulatory mechanism for glandular cells is partially conserved, it is largely species‐specific.^[^ ^107^ ^]^ Further studies revealed that CsMYB36 activates CsGL1 expression to maintain reactive oxygen species (ROS) homeostasis, thereby driving the transition of trichome cells from division to differentiation.^[^ ^108^ ^]^ The functional conservation of HD‐ZIP I genes is further highlighted by the role of CsLMI1, a homolog in citrus, which is essential for secretory cavity formation. Collectively, these findings demonstrate that the HD‐ZIP I family plays a highly conserved, yet functionally versatile, role in glandular cell differentiation.^[^ ^109^ ^]^

However, it remains unclear whether this mechanism operates in other species. In tobacco (*Nicotiana tabacum*), another Solanaceae species, two HD‐Zip IV genes, NtHD9 and NtHD12, have recently been reported to control glandular head formation.^[^ ^105^, ^110^ ^]^ Their functions appear linked to JA signaling, suggesting environmental responsiveness. In *Artemisia annua*, the MADS‐box factor AaSEPALLATA1 (AaSEP1) integrates jasmonate and light‐induced GT initiation; its overexpression substantially increases GT density.^[^ ^73^ ^]^ In *Artemisia annua*, another study reveals that AaHD1‐AaMYB16 module also regulates GT initiation via JA signaling.^[^ ^57^ ^]^ Interestingly, another MYB transcription factor AaMYB5 is found to competitively bind to AaHD1, thus repressing the activation of downstream gene AaGSW2, a WRKY transcription factor, and inhibiting glandular cell formation.^[^ ^57^ ^]^ In addition to AaMYB5, a R2R3‐MYB protein named TLR1 has also been found to interact with AaWOX1 to repress the formation of glandular cells in *Artemisia annua*.^[^ ^111^ ^]^ Additionally, the AP2/ERF gene AaWIN1 has also been found to serve as a positive GT regulator, and AaMIXTA1 interacts with AaWIN1 to regulate GT initiation in *Artemisia annua*.^[^ ^112^ ^]^ While informative, these findings rely heavily on overexpression evidence. Loss‐of‐function genetics are needed to validate their roles in GT development in *A. annua* and determine conservation across species.

Furthermore, accumulating evidence indicates that the AP2/ERF family plays a crucial role in glandular development. In *Artemisia annua*, the AP2/ERF protein AaWIN1 was shown to positively affect glandular trichome development, where it interacts with AaMIXTA1 to coordinately regulate GT initiation.^[^ ^112^ ^]^ In tomato, the AP2/ERF transcription factor LFS decides the early developmental fate of glandular trichomes, and its knockout leads to the complete absence of glandular cells. This mechanism appears conserved across species, as demonstrated in citrus where CsDRNL, a LFS homolog, is involved in secretory cavity formation and its knockout results in a complete loss of oil cells.^[^ ^113^ ^]^

The AP2/ERF transcription factor GhERF105 regulates gland pigmentation and VIGS‐mediated gene silencing dramatically reduces gland numbers in cotton leaves.^[^ ^113^ ^]^ Interestingly, map‐based cloning also identified AP2/ERF transcription factor GoNe as the key regulator of the formation of both floral and extrafloral nectaries (Figure 6).^[^ ^114^ ^]^ In addition to AP2 genes, other gene families have also been found to affect gland formation in cottons. In a glandless cotton mutant, map‐based cloning identified Gossypium PIGMENT GLAND FORMATION GENE (GoPGF), a helix‐loop‐helix transcription factor, as the causative gene.^[^ ^115^ ^]^ Silencing GoPGF yields a glandless phenotype, confirming its role as a positive regulator.^[^ ^115^ ^]^

Similarly, in tomato, the bHLH factor SlMYC1 was found to affect glandular trichome formation.^[^ ^35^ ^]^ CRISPR‐Cas9 knockout of SlMYC1 reduces type VI trichome density, while overexpression increases it.^[^ ^35^, ^116^, ^117^ ^]^ In many other systems, MYC genes are reported to mediate JA signaling. In glandular trichome development, SlMYC1 was found to form a regulatory module with a GRAS family transcription factor SlGRAS9.^[^ ^117^ ^]^ This GRAS protein negatively regulates SlMYC1 transcription, and consistent with this, SlGRAS9 knockout increases type VI GT density.^[^ ^117^ ^]^ Involvement of GRAS gene in gland development is not restricted to tomato. In consistent with this, a map‐based cloning in cotton identified a GRAS transcription factor called GoSPGF as responsible for stem‐specific glandless traits.^[^ ^118^ ^]^ GoSPGF was shown to directly activate GoPGF, and together they form a hierarchical transcriptional network with GhWRKYs to promote the Pigment gland development in cotton.^[^ ^119^ ^]^

## 6 Conclusions and Future Perspectives

Plant trichomes represent a remarkable example of how plants have evolved sophisticated epidermal structures to adapt to their environments. This review has synthesized recent advances revealing a significant divergence in the developmental programming of trichomes, moving beyond the canonical MYB‐bHLH‐WD40 (MBW) module established in *Arabidopsis*. A central theme emerging from studies on multicellular and glandular trichomes is the pivotal role of HD‐Zip IV transcription factors, which can act through dose‐dependent mechanisms. The discovery of intricate regulatory circuits for trichome type specification and glandular cell formation underscores the layered complexity of trichome development. Despite extensive research on trichome development across species, significant questions remain.

The evolutionary pathways leading to the distinct regulatory mechanisms of unicellular trichomes (the MBW module in *Arabidopsis*) versus multicellular/glandular trichomes (the HD Zip IV module in tomato) remain enigmatic. It is unclear how the key components of these regulatory modules were replaced or modified to adapt to various environments. Comparative genomics and phylogenomic analyses across a broader range of plant taxa may help reconstruct the evolutionary history of the trichome regulatory network.

Trichome is initiated as the protrusion of epidermal cells. A similar developmental process shapes the conical cells typical of the petal epidermis in around 79% of angiosperms, and the conserved role of MIXTA transcription factors in conical cell development is well‐established. In contrast, the MBW complex and members of the HD‐ZIP IV family appear to play a central role in initiating trichomes with more complex morphologies. Classical genetics in *Arabidopsis* established the paradigm of the MBW complex in regulating trichomes via the HD‐ZIP IV gene GLABRA2 (GL2). However, in some other species, trichome fate is directly controlled by HD‐ZIP IV genes, with the MBW complex losing its dominant role in trichome development; in these species, the regulatory network hierarchy may have been rewired. Furthermore, in monocots, the trichome regulatory network seems to be dominated by SPL regulators, but whether they replace the conserved genes of MBW complex or HD Zip family requires further exploration. Notably, WOX3B is a key factor in trichome formation in both monocots and eudicots. One possibility is that in monocots, changes in cis‐regulatory elements allowed SPL regulators to take over and rewire the regulatory network.

Research on tomato trichomes provides relatively clear evidence: the HD‐ZIP IV member Wo precisely regulates the division and differentiation of trichome types through a concentration gradient. This concentration‐dependent feedback mechanism ensures the spatiotemporal patterning of trichome cell regulation. Interestingly, multicellular non‐glandular trichomes and glandular trichomes share the same initial fate determinants, but the developmental trajectories of their apical cells differ. The specialization of apical cells in glandular trichomes is specifically regulated by genes related to gland formation. These gland regulatory genes appear to be highly conserved in angiosperms such as cotton and citrus. However, the downstream targets of these conserved transcription factors appear to differ significantly across species, leading to diversity in trichome morphology and function.

Functional replacement or alteration of key trichome regulatory genes seems to be relatively easily achieved during evolution. For instance, in *Artemisia annua*, the MIXTA transcription factor interacts with an HD‐ZIP IV transcription factor to co‐regulate trichome initiation; in tomato, MIXTA acts as a downstream gene of the HD‐ZIP IV factor; and in Nigella petals, HD‐ZIP IV is still involved in the formation of long and short trichomes, while MIXTA retains only a role in cuticle modification of epidermal cells. This demonstrates that the differential recruitment of conserved transcription factors and the rewiring of regulatory networks are important mechanisms driving the evolution of trichome diversity.

The second challenge lies in understanding how environmental signals are integrated with the trichome developmental programs to precisely trigger and modulate the trichome development in different conditions. It remains elusive how environment‐induced plant hormones, such as jasmonic acid (JA), crosstalk with core multicellular trichome regulators like Wo and MTR, and how they influence the concentration thresholds for Wo activity. Third, a crucial yet unresolved question is how the developmental program is coupled with the activation of metabolic pathways. For glandular trichomes, identifying the transcription factors and their direct targets that can directly bridge development and metabolism is essential for the applications of trichomes in future synthetic biology. This would enable us to design metabolic factories, rather than merely increasing trichome density. Forth, an intriguing related aspect of glandular trichomes is the cellular mechanism underlying the production, transport, and storage of metabolic products. As metabolic factories, these cells employ specialized storage strategies. While previous cellular studies have largely focused on *Cannabis sativa*, recent work has identified a novel compartmentalization structure in cucumber glandular cells. Silica deposited on cucumber fruit surfaces forms a white powder, known as “bloom”, which is transported to and stored in glandular trichomes.^[^ ^120^ ^]^ In a bloomless mutant, the MYB family gene MYB36 was found to control the formation of a lignin‐based “neck strip” in glandular cells—an extracellular barrier essential for silica accumulation. Interestingly, a similar structure has been identified in the chalazal region of *Arabidopsis* seeds, suggesting this mechanism may be conserved across different tissues and plant species.^[^ ^121^ ^]^

## Conflict of Interest

The authors declare no conflict of interest.

## Author Contributions

M.L., Y.H., and S.W. wrote the manuscript. Y.D. and Y.H. drew the graphic figures.
